# Assessment of the conjunctival microcirculation for patients presenting with acute myocardial infarction compared to healthy controls

**DOI:** 10.1038/s41598-021-87315-7

**Published:** 2021-04-07

**Authors:** Paul F. Brennan, Andrew J. McNeil, Min Jing, Agnes Awuah, Julie S. Moore, Jonathan Mailey, Dewar D. Finlay, Kevin Blighe, James A. D. McLaughlin, M. Andrew Nesbit, Emanuele Trucco, Tara C. B. Moore, Mark S. Spence

**Affiliations:** 1 Department of Cardiology, Royal Victoria Hospital, Belfast Health and Social Care Trust, Belfast, UK; 2 Biomedical Sciences Research Institute, Ulster University, Coleraine, UK; 3 VAMPIRE project, Computing (SSEN), University of Dundee, Dundee, UK; 4 Nanotechnology and Integrated Bioengineering Centre (NIBEC), Ulster University, Jordanstown, UK

**Keywords:** Anatomy, Cardiology, Medical research, Pathogenesis, Risk factors

## Abstract

Microcirculatory dysfunction occurs early in cardiovascular disease (CVD) development. Acute myocardial infarction (MI) is a late consequence of CVD. The conjunctival microcirculation is readily-accessible for quantitative assessment and has not previously been studied in MI patients. We compared the conjunctival microcirculation of acute MI patients and age/sex-matched healthy controls to determine if there were differences in microcirculatory parameters. We acquired images using an iPhone 6s and slit-lamp biomicroscope. Parameters measured included diameter, axial velocity, wall shear rate and blood volume flow. Results are for all vessels as they were not sub-classified into arterioles or venules. The conjunctival microcirculation was assessed in 56 controls and 59 inpatients with a presenting diagnosis of MI. Mean vessel diameter for the controls was 21.41 ± 7.57 μm compared to 22.32 ± 7.66 μm for the MI patients (p < 0.001). Axial velocity for the controls was 0.53 ± 0.15 mm/s compared to 0.49 ± 0.17 mm/s for the MI patients (p < 0.001). Wall shear rate was higher for controls than MI patients (162 ± 93 s^−1^ vs 145 ± 88 s^−1^, p < 0.001). Blood volume flow did not differ significantly for the controls and MI patients (153 ± 124 pl/s vs 154 ± 125 pl/s, p = 0.84). This pilot iPhone and slit-lamp assessment of the conjunctival microcirculation found lower axial velocity and wall shear rate in patients with acute MI. Further study is required to correlate these findings further and assess long-term outcomes in this patient group with a severe CVD phenotype.

## Introduction

Globally cardiovascular disease (CVD) is a leading cause of morbidity and mortality with an estimated 17.7 million people dying from CVD each year^1^. CVD also represents a substantial economic burden and in Northern Ireland alone, it is estimated that around £400 million per year is spent on CVD^2^. Cardiovascular screening and primary prevention are important elements of the healthcare system. CVD risk models such as the Q-Risk 3 score provide a 10-year estimate of the likelihood of a patient sustaining a major adverse cardiovascular event (MACE) occurring e.g. myocardial infarction (MI), cerebrovascular accident (CVA)^3,4^.

Microcirculatory dysfunction represents the earliest stages of CVD^5^. Microvascular networks that are readily-accessible for evaluation include the retinal circulation, sublingual circulation and nail-fold circulation^6–8^. The conjunctival microcirculation gains its blood supply from the anterior ciliary branch of the ophthalmic artery^9^. Conjunctival microcirculatory parameters, namely diameter (D), axial velocity (Va), blood volume flow (Q)and wall shear rate/stress (WSR/WSS) have previously been assessed using a non-invasive combination of a slit-lamp biomicroscope and digital charge-coupled device (CCD) camera^10–16^. The study of the conjunctival microcirculation, using these methods, has been applied to patients at high CVD-risk e.g. post CVA, diabetic retinopathy^17,18^. Correlation between conjunctival microcirculatory abnormalities and CVD risk calculation, using the Framingham Risk Score has also been reported^19,20^. Haemoglobin video imaging (HVI) has recently been described for assessing the branching patterns within the conjunctival circulation^21^.

Assessment of the conjunctival microcirculation in patients with acute MI, has not been described. A prior rabbit model^22^, however, demonstrated a relationship between conjunctival venous diameter velocity, blood volume flow and the administration of either the potent vasoconstrictor alpha-adrenergic agonist phenylephrine or the negatively inotropic beta-adrenergic blocker esmolol. Nail-fold capillary diameters have been shown to be more dilated in patients with chronic heart failure (CHF) compared to controls^23^. These responses of the microcirculation to alterations in vasoreactivity, cardiac inotropy and heart failure status could all potentially be encountered in patients with acute MI.

We recently reported the feasibility of assessing the conjunctival microcirculation using an iPhone 6s (Apple, USA) and slit-lamp biomicroscope in a group of low CVD-risk controls^24^. The purpose of this study was to quantifiably assess the conjunctival microcirculation of patients that had recently suffered an acute type 1 MI, based on the 4th universal definition of MI compared to a group of age- and sex-matched controls^25^.

## Methods

### Study design

We conducted a prospective study (Integrated Research Application System study number 166742) of inpatients who had suffered a recent myocardial infarction compared to a group of low CVD risk controls at the Royal Victoria Hospital, Belfast, United Kingdom. All subjects were screened and provided with verbal and written information and consented prior to study enrolment. Informed consent was obtained from all participants. Participants were recruited to the study in accordance with the Declaration of Helsinki. The experimental protocol was approved by the Research Ethics committee in the Belfast Health and Social Care Trust (BHSCT) and Ulster University (UU).

Criteria for the MI patients included that they were currently an inpatient with acute type 1 MI, not pregnant and greater than 17 years age. Key inclusion criteria for the control patients was that they were of low CVD risk and did not have prior history of coronary artery disease, MI, stroke, diabetes or uncontrolled systemic hypertension. Exclusion criteria for both groups included inability to consent, a history of recent conjunctival inflammation, prior refractive surgery, use of ocular medications (excluding artificial tears) and current use of contact lenses. In addition to the exclusion screening questionnaire all patients were examined by a clinical optometrist at the time of conjunctival imaging to assess for active signs of inflammation or dry eyes.

Baseline characteristics and quantitative conjunctival microcirculatory parameters were compared between both patient groups. The Q-Risk 3 score was calculated to estimate the 10-year risk of MACE for the control group.

The definitions of ST elevation and Non-ST elevation MI (STEMI, NSTEMI) were as described in the respective European Society of Cardiology guidelines^26,27^.

Baseline clinical data and characteristics were obtained using the recruitment questionnaire, the inpatient clinical notes (if an acute MI patient), the hospital cardiology database (Cardiovascular Information System Tomcat, Phillips, Eindhoven, Netherlands) and the patient’s Northern Ireland Electronic Care Record (NIECR). Long-term clinical outcomes were determined using NIECR.

### Conjunctival assessment

**Figure 1:**
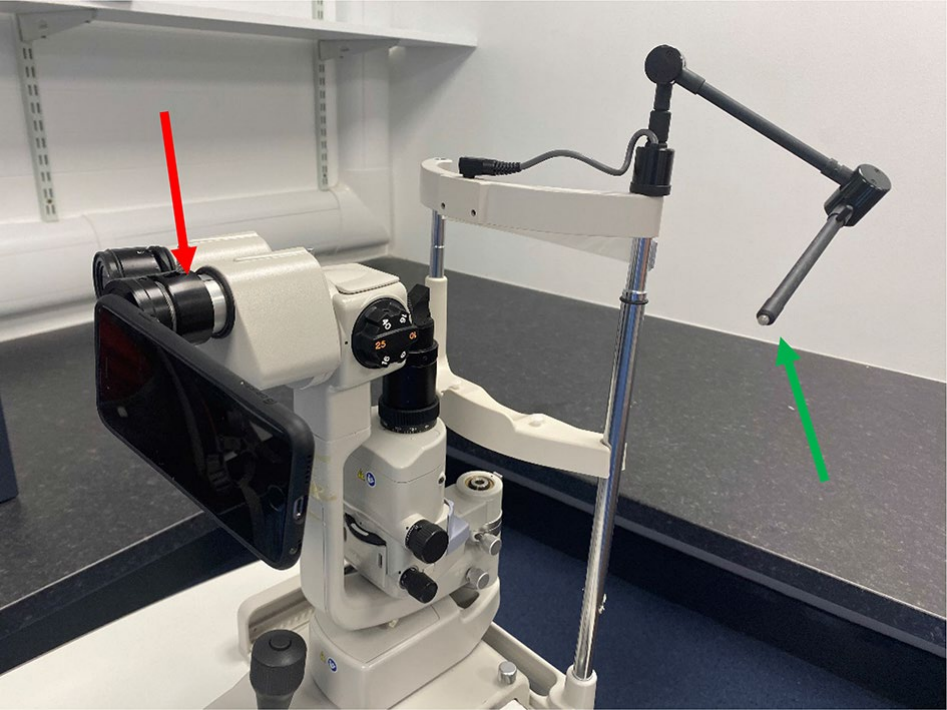
The iPhone 6s, TopCon SL-D4 imaging system with the Zarf bespoke adapter (red arrow) and TopCon external fixation target (green arrow).

Conjunctival microcirculation image acquisition for both eyes was performed under the same settings for all patients. Using our previously reported methods^24^ we acquired images using a Topcon SL-D4 (Topcon Medical Systems Inc., USA), an iPhone 6s smartphone (Apple, Inc, USA) and a bespoke adapter (Zarf Enterprises Inc., USA) as illustrated in Fig. 1. The optimal configuration was set at a resolution of 1920 × 1080 pixels, captured at 60 frames per second. Using the third-party application “ProMovie Recorder” (http://www.promovieapp.com) we locked the video zoom setting at magnification 2×, providing a 1:1- pixel mapping of the camera sensor at 1080 pixel resolution. We acquired 5–10 s videos of the conjunctival microcirculation medial and lateral to the iris, this generating four videos per subject. An external fixation target was used to minimise blinking and eye motion. A processed stabilised video of the left nasal conjunctival microcirculation is provided in Supplementary Video file 1.

We quantifiably assessed diameter (D), axial velocity (Va), blood volume flow (Q) and wall shear rate (WSR) in vessels with observable flow.

MALTAB R2019b (MathWork, USA) was used for programming. The sharpest frame in the sequence was automatically selected as a reference frame and all other frames registered to it. The Matlab function imregister.m was used for automatic registration. Automatic vessel filtering was implemented using Matlab code available in^28^ Vessel centreline extraction and branch/end-point detection was automatically applied to the image using Matlab function bwmorph.m.

The Euclidean Distance Transform (EDT) principle was used to calculate vessel diameter (D). The value at each pixel of EDT map was automatically calculated based on the Euclidean distance between the pixel and its nearest nonzero pixel in the binary vessel image. The vessel centreline was used to obtain the central EDT values and radius along the vessel axis. The final vessel diameter estimation is provided by averaging the diameters found along the length of the vessel.

Axial velocity was estimated based on the spatial–temporal image (STI) obtained from the vessel segment and continuous wavelet transform (CWT) method, with the change in STI intensity reflecting erythrocyte movement through the vessel. The (CWT) has been applied as a spatio-temporal filter for motion capture for 1-dimension plus time (1D + T) signals^29–31^. The STI is a time sequence signal denoted as ***I(x,t),*** where each value in the image represents the intensity at the position *x* in the corresponding vessel centreline and time point *t*. The change in STI intensity reveals the blood flowing through the vessel within the given time (or video frames) and as described previously this was performed semi-automatically^24^.

The blood volume flow rate (Q) was automatically calculated based on the principles defined in previous works^11,24^ by the product of the cross-sectional velocity ${V}_{s}$ and the cross-sectional area (assuming a circular cross-section (1)):

1 $$Q= {V}_{s}\frac{\pi {D}^{2}}{4}$$

The wall shear rate (WSR) was calculated by dividing the cross-sectional velocity by the diameter as evidenced in (2)

2 $$WSR= \frac{8{V}_{s}}{D}$$

For microvessel diameters less than approximately 20 μm, a velocity profile cannot be used in the ordinary sense in order to estimate ${V}_{s}$^32^. Therefore, ${V}_{s}$ is obtained based on a profile factor function that has been previously described in other work^33^, in which the relationship between the cross-sectional velocity ${V}_{s}$ and axial velocity ${V}_{a}$ is represented as:

3 $${V}_{s} = \left\{ {\begin{array}{ll}
{{V}_{a}}& \quad {when \; D/{D}_{c}\le 0.6,}\\
{\frac{{{V_a}}}{{1.58\left( {1 - {e^{ - \sqrt {{{2D} \mathord{\left/
 {\vphantom {{2D} {{D_c}}}} \right.
 \kern-\nulldelimiterspace} {{D_c}}}} }}} \right)}}}, & \quad {when \;  D/{D}_{c} > 0.6.}
\end{array}} \right.$$

where ${D}_{c}$ is the size of the average human erythrocyte diameter that is 7.65 μm as described in^33^.

**Figure 2:**
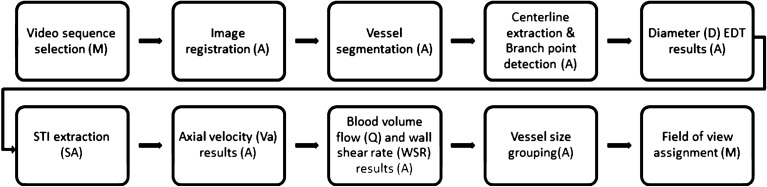
Flow-chart illustrating the individual processing stages from image registration to results output. Each step is labelled as automatic (A), manual (M) or semiautomatic (SA).

Figure 2 is a flow-chart illustrating the processing stages.

Methodology repeatability was assessed by one observer analysing the video sequences for 5 control subjects twice, blinded to the clinical and demographic details of each subject. The repeated measurements were obtained from 38 vessel segments (5–9 per subject) and sample size was similar to other well-established conjunctival analysis work^16^. Differences in the four main measured parameters (D, Va, Q, WSR) were compared. Coefficients of repeatability (CR) were calculated using conventional approaches^33^. The mean difference for D was − 0.01 ± 0.04 μm, CR 0.08 μm (95%CI − 0.09 μm to 0.07 μm) and for Va was 0.002 mm/s ± 0.01 mm/s, CR 0.02 mm/s (95%CI − 0.02 mm/s to 0.02 mm/s). The mean difference for Q was 0.03 ± 2.14 pl/s, CR 4.19 pl/s (95%CI − 4.1 pl/s to 4.2 pl/s) and for WSR was 0.78 ± 4.17 s^−1^, CR 8.18 s^−1^ (95%CI − 7.4 to 9.0 s^−1^). These results supported acceptable repeatability of vessel segment analysis using our current technical approach.

To overcome the heterogenous anatomical properties of the conjunctival microcirculation we applied a vessel grouping classification to our results based on vessel diameter i.e. group 1 (D < 11 μm), group 2 (D 11–16 μm), group 3 (D 16–22 μm) and group 4 (D > 22 μm)^10,24^. Vessels were not separated into venules or arterioles and results are provided for all vessels.

### Statistical analysis

For statistical analysis SPSS for Apple iOS version 25 (property of IBM) was used. Continuous variables were described using the mean, standard deviation of the mean and 95% confidence intervals (CI). The median was applied if the continuous variable was not normally distributed. Kolmogorov–Smirnov testing was used to assess normality of the continuous variables. Categorical variables were expressed as a number and percentage of the total category number to which the variable belonged.

Normally distributed variables were compared between the two populations using the independent-samples t-test. Non-normally distributed continuous variables were compared using non-parametric tests e.g. Mann–Whitney U test. Categorical comparisons were made between the two groups using Pearson Chi-Square or Fisher’s exact test.

A one-way analysis of variance (ANOVA) was used to compare differences between the two groups based on the vessel group classifications and the fields of view imaged, followed by post-hoc testing where indicated to assess the origin of the statistical significance. Bonferroni post-hoc testing was applied if normal variance was assumed and Games-Howell if normal variance could not be assumed.

An α-level of less than 0.05 was determined to be of statistical significance. As this was an exploratory pilot study a formal power calculation was not performed. Results are described based on the number of vessel segments. A total of 4163 vessel segments were analysed and reported in the results section (controls 1904 total, 34 per patient vs MI 2259 total, 38 per patient) providing a large number of vessels for comparison. The parameters for each study group from all vessel segments across the four videos were averaged for each patient prior to overall analysis. When vessel groups were compared (1–4), however, each parameter was averaged for all vessel segments falling within that vessel group.

## Results

### Baseline characteristics

Between the 31st January 2018 and 15th March 2019, 115 patients were recruited to this study. 48.7% (56/115) patients were healthy controls and 51.3% (59/115) were patients post-acute MI. The mean age for the control population was 53 ± 10 years compared to 57 ± 12 years for the acute MI patients (p = 0.07). 68% (38/56) the control population were male compared to 80% (47/59) of the acute MI patients (p = 0.15). The MI patients had a higher proportion of diabetes mellitus, systemic hypertension and smoking. The baseline Q-Risk 3 score for the controls was 8.1 ± 7.6%. Baseline characteristics are summarised in Table 1.

**Table 1:** Summary of baseline characteristics for the healthy control and acute MI populations.

Baseline characteristic	Control (n = 56)	MI (n = 59)	p value
Age, years ± SD	53 ± 10	57 ± 12	0.07
Male sex n (%)	38 (68)	47 (80)	0.15
Q-Risk 3 score ± SD (%)	8.1 ± 7.6	Not applicable	
Clinical IHD n (%)	0	11 (18.6)	** < 0.001**
Prior MI n (%)	0	8 (13.6)	**0.006**
Prior stroke n (%)	0	1 (1.7)	1
Hypertension n (%)	7 (12.5)	21 (35.6)	**0.004**
Diabetes mellitus n (%)	0	12 (20.3)	**0.002**
Dyslipidaemia n (%)	11 (19.6)	25 (42.4)	**0.009**
Smoking history n (%)	21 (37.5)	36 (61)	**0.01**
COPD n (%)	4 (7.1)	2 (3.4)	0.43
Creatine clearance, ml/min ± SD	84 ± 29	91 ± 37	0.56
Haemoglobin, g/l ± SD	146 ± 11	142 ± 15	0.08
Haematocrit, l/l ± SD	0.43 ± .03	0.42 ± .04	0.29
Platelet count, 10^x9^/l ± SD	263 ± 43	259 ± 64	0.27

Bold values are statistically significant (p < 0.05).

*IHD* ischaemic heart disease, *MI* myocardial infarction, *COPD* chronic obstructive pulmonary disease.

At the time of recruitment both groups were normotensive, with the control population having a significantly higher systolic (129 ± 16 mmHg vs. 121 ± 17 mmHg, p = 0.01) blood pressure and higher diastolic (77 ± 10 mmHg vs. 74 ± 11 mmHg, p = 0.10) blood pressure, but this did not reach statistical significance. This is explained by the appropriate administration of blood pressure (BP)-lowering medications, either newly started or long-term, for 95% of the MI population at the time of recruitment and patient medications are summarised in Table 2.

**Table 2:** Pharmacological therapy at the time of recruitment.

Medication	Control population (n = 56)	MI population (n = 59)	p value
Aspirin, n (%)	5 (8.9)	58 (98.3)	** < 0.001**
Ticagrelor, n (%)	0	52 (88.1)	** < 0.001**
Clopidogrel, n (%)	0	2 (3.4)	0.50
Prasugrel, n (%)	0	0	n/a
ACE inhibitors, n (%)	5 (8.9)	41 (69.5)	** < 0.001**
Angiotensin receptor blockers, n (%)	1 (1.8)	7 (11.9)	0.10
Mineralocorticoid antagonists, n (%)	0	11 (18.7)	**0.003**
Beta-blockers, n (%)	3 (5.4)	53 (89.8)	** < 0.001**
Any BP-lowering medication, n (%)	7 (12.5)	56 (95)	** < 0.001**
Statins, n (%)	8 (14.3)	55 (93.2)	** < 0.001**

Bold values are statistically significant (p < 0.05).

Within the MI group 21 (36%) had STEMI and 38 (64%) NSTEMI. There were no differences in mean age or sex of either MI group. The median time to recruitment post MI event was 1 day (interquartile range IQR 1–3, overall range day 0–22). 81% patients were recruited within 3 days of their MI, 91.5% within the first week and 98.3% within 2 weeks. 49 (83%) of the MI patients had percutaneous coronary intervention (PCI), 6 (10%) had coronary artery bypass grafts (CABG) and 4 (7%) patients were medically managed without coronary revascularisation. 28 (47%) of MI patients were recruited prior to coronary revascularisation with the remaining 31 (53%) of MI patients recruited after revascularisation. The breakdown of STEMI, in order of most to least frequent and based on the presenting electrocardiogram’s (ECG) suggested territory of infarction, was 47.7% (n = 10) inferior, 33.3% (n = 7) anterior, 9.5% (n = 2) lateral and 9.5% (n = 2) posterior/inferolateral.

Left ventricular systolic dysfunction, based on a left ventricular ejection fraction < 50%, was observed in 46% (n = 27) MI patients on their pre-discharge transthoracic echocardiogram (TTE). 8.5% (n = 5) patients had severe left ventricular systolic dysfunction (ejection fraction < 35%) on TTE. There was no in-hospital mortality, further MI or stroke. No patients had an implantable cardioverting defibrillator (ICD) or permanent pacemaker (PPM) implanted. The STEMI group had a statistically higher TnThs compared to the NSTEMI group (3645 ± 3438 mmol/l vs. 527 ± 1077 mmol/l, p = 0.001) but creatinine clearance, Nt-proBNP and baseline lipid profiles did not differ, significantly, between the two groups. Baseline comorbidities were similar between the STEMI and NSTEMI group and are summarised in Table 3.

**Table 3:** Baseline characteristics and biomarkers for the STEMI and NSTEMI populations.

Baseline characteristic	STEMI (n = 21)	NSTEMI (n = 38)	p value
Age, years ± SD	55 ± 13	59 ± 11	0.20
Male sex, n (%)	17 (81)	30 (79)	1
Creatine clearance, ml/min ± SD	99 ± 50	89 ± 24	0.41
Diabetes mellitus, n (%)	2 (9.5)	10 (26.3)	0.18
Hypertension, n (%)	5 (23.8)	16 (42)	0.25
LVEF < 50%, n (%)	12 (57)	15 (38.5)	0.42
TnThs, mmol/l ± SD	3645 ± 3438	527 ± 1077	0.001
NT-proBNP, ng/L ± SD	1288 ± 1377	825 ± 1190	0.22
LDL, mmol/l ± SD	3.2 ± 1.7	2.6 ± 1.3	0.16
Cholesterol/HDL ratio ± SD	4.2 ± 0.4	4.2 ± 1.6	0.83
HbA1c, mmol/l ± SD	41 ± 5	49 ± 17	0.09
Time in hospital, days (IQR)	4 (2–6.5)	9 (2–10.5)	0.17

*SD* standard deviation, *STEMI* ST segment elevation myocardial infarction, *NSTEMI* non ST segment elevation myocardial infarction, *TnThs* high-sensitivity troponin T, *NT-proBNP* N terminal pro brain natriuretic peptide, *LDL* low density lipoprotein, *HDL* high density lipoprotein, *HbA1c* glycated haemoglobin, *LVEF* left ventricular ejection fraction, *IQR* interquartile range.

STEMI patients had a shorter total admission duration than NSTEMI patients (4.2 ± 3 days vs. 9.2 ± 13 days, p = 0.17). STEMI patients were treated via our regional primary PCI pathway with immediate revascularisation whereas the mean time to NSTEMI patient revascularisation was 5.6 ± 7 days. The mean time for NSTEMI patients to revascularisation with PCI was 2 ± 2 days compared to 18 ± 8 days for CABG (p < 0.001) and the PCI patients also had significantly shorter stays post revascularisation (2.3 ± 3 days vs. 13 ± 12 days, p < 0.001).

Repeat revascularisation occurred in 17% (n = 10) patients during a median follow-up of 361 days. The majority of repeat revascularisation was planned (70%, n = 7). Unplanned revascularisation occurred in 30% (n = 3) of the MI repeat revascularisation group. Culprit vessel revascularisation occurred in 30% (n = 3) of this group. There were no cases of stent thrombosis during follow-up.

Only one patient was readmitted with HF presentation within 1 year. There was no cardiac mortality during follow-up. One MI patient died of lung adenocarcinoma (diagnosed after recruitment) during follow-up.

### Conjunctival analysis

Conjunctival microvascular videos were captured for all 115 patients. There were no reported complications or adverse outcomes associated with image acquisition. Processing of images and subsequent microvascular quantitative assessment was performed in an independent laboratory, blinded to the patient baseline characteristics and history to avoid bias.

A total of 4163 (control 1904 total, 34 per patient vs. MI 2259 total, 38 per patient) vessel segments were analysed for the two populations, with no significant differences in the mean number of vessel segments analysed per patient group (p > 0.05).

The mean vessel diameter for the controls was 21.41 ± 7.57 μm (95% CI 21.08–21.76 μm) which was significantly lower than the 22.32 ± 7.66 μm (95% CI 22–22.64 μm) for the MI patients (p < 0.001). The mean axial velocity (Va) for the MI population was 0.49 ± 0.17 mm/s (95% CI 0.49–0.50 mm/s) which was significantly lower than the control populations mean Va of 0.53 ± 0.15 mm/s (95% CI 0.52–0.53 mm/s (p < 0.001)).

Overall mean blood volume flow (Q) did not differ significantly between the control and MI groups (153 ± 124 pl/s (95% CI 147–157 pl/s) vs. 154 ± 125 pl/s (95% CI 149–159 pl/s), p = 0.84). Wall shear rate (WSR) was significantly lower in the MI population, compared to the healthy controls (control 162 ± 93 s^−1^ (95% CI 118–166 s^−1^) vs. MI 145 ± 88 s^−1^ (95% CI 141–149 s^−1^), p < 0.001). Table 4 summarises the differences between the two study groups.

**Table 4:** Summary of conjunctival microcirculatory parameters for all vessel sizes for both populations.

Haemodynamic parameter	Controls (n = 56)	MI patients (n = 59)	p value
D, μm ± SD	21.43 ± 7.57	22.32 ± 7.66	** < 0.001**
Va, mm/s ± SD	0.53 ± 0.15	0.49 ± 0.17	** < 0.001**
Q, pl/s ± SD	153 ± 124	154 ± 125	0.84
WSR, s^−1^ ± SD	162 ± 93	145 ± 88	** < 0.001**

Bold values are statistically significant (p < 0.05).

*D* diameter, *SD* standard deviation, *Va* axial velocity, *Q* blood volume flow, *WSR* wall shear rate, *MI* myocardial infarction.

Microcirculatory parameters were sub-analysed between the two groups based on the four vessel size groupings described above. The largest diameter vessels (group 4, > 22 μm) were the most frequently observed vessels with vessel frequency decreasing from group 4 down to group 1.

Axial velocity (Va) was lower for the MI patients through all vessel size groups, meeting statistical significance for groups 2–4. Blood volume flow (Q) was also lower for MI patients in all vessel size groups and this met statistical significance for the most numerically abundant vessels i.e. groups 3 and 4. Wall shear rate (WSR) was consistently lower for the MI patients for all vessel groups and met statistical significance for groups 2–4. Table 5 is a summary of the microcirculatory comparisons for each of the four vessel size groups.

**Table 5:** Comparison of conjunctival microcirculatory parameters based on vessel group.

Haemodynamic parameter	Control group (n = 56)	MI population (n = 59)	p value
**Group 1 (< 11 μm)**	**No. vessels 165**	**No. vessels 178**	
D, μm ± SD	9.12 ± 1.33	9.04 ± 1.36	0.64
Va, mm/s ± SD	0.49 ± 0.14	0.46 ± 0.15	0.08
Q, pl/s ± SD	26 ± 10	24 ± 10	0.15
WSR, s^−1^ ± SD	357 ± 132	339 ± 128	0.22
**Group 2 (11–16 μm)**	**No. vessels 325**	**No. vessels 324**	
D, μm ± SD	13.55 ± 1.47	13.70 ± 1.35	0.205
Va, mm/s ± SD	0.49 ± 0.14	0.46 ± 0.16	**0.02**
Q, pl/s ± SD	54 ± 19	52 ± 21	0.13
WSR, s^−1^ ± SD	221 ± 70	205 ± 75	**0.005**
**Group 3 (16–22 μm)**	**No. vessels 512**	**No. vessels 571**	
D, μm ± SD	19.16 ± 1.78	19.26 ± 1.72	0.382
Va, mm/s ± SD	0.51 ± 0.14	0.47 ± 0.16	** < 0.001**
Q, pl/s ± SD	105 ± 35	99 ± 39	**0.001**
WSR, s^−1^ ± SD	152 ± 45	140 ± 49	** < 0.001**
**Group 4 (> 22 μm)**	**No. vessels 902**	**No. vessels 1186**	
D, μm ± SD	27.81 ± 4.91	28.14 ± 4.93	0.06
Va, mm/s ± SD	0.55 ± 0.16	0.52 ± 0.17	** < 0.001**
Q, pl/s ± SD	239 ± 129	228 ± 128	**0.01**
WSR, s^−1^ ± SD	111 ± 35	102 ± 37	** < 0.001**

Bold values are statistically significant (p < 0.05).

*D* diameter, *SD* standard deviation, *Va* axial velocity, *Q* blood volume flow, *WSR* wall shear rate, *MI* myocardial infarction.

The conjunctival measurements were compared between the STEMI and NSTEMI patient groups. Despite the differences in presentation, myocardial biomarkers and prevalence of left ventricular systolic dysfunction there were no significant differences between the two groups with respect to any of the conjunctival measurements as summarised in Supplementary Table 1.

**Figure 3:**
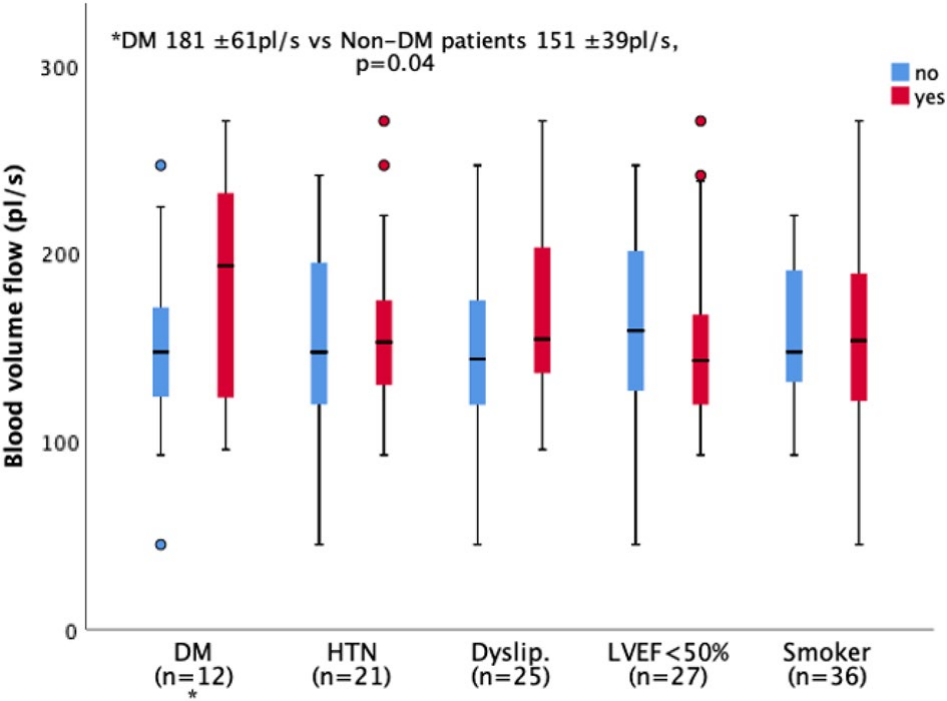
Sub-group analysis of MI patients with respect to microvessel blood volume flow (Q). n denotes the number of “yes” for each sub-group. *DM* diabetes mellitus, *HTN* hypertension, *Dyslip.* dyslipidaemia, *LVEF < 50%* left ventricular ejection fraction less than 50%, *Smoker* active smoker or ex-smoker vs no history smoking.

Within the MI population sub-group analysis of specific comorbidities the only significant difference in microcirculatory parameters was a higher Q (181 ± 61 pl/s vs 151 ± 39 pl/s, p = 0.04) in diabetic (DM) patients (n = 12) compared to non-diabetics (n = 47) as presented in Fig. 3.

No significant differences were noted for the remainder of the microcirculatory parameters on subgroup analysis of smoking st.atus, impaired LV systolic function (LVEF < 50%), systemic HTN and hypercholesterolaemia. Supplementary Figure 1a–c illustrate the microcirculatory parameters sub-group analysis.

## Discussion

Our study demonstrated differences in conjunctival microcirculatory parameters for a group of high CVD risk patients post-MI compared to a group of healthy volunteers. The physiological changes and endothelial dysfunction, associated with atherosclerosis and CVD, typically manifest earliest in the microvasculature within the body^5^. The progressive process which leads to myocardial ischaemia is known as the ischaemic cascade^34^. This process begins with localised metabolic and endothelial function changes in the coronary circulation. Perfusion defects typically occur next in this cascade, followed by cardiac myocyte dysfunction and changes in the patient ECG which can result in angina or MI.

There was a significant reduction in Va for patients with history of recent acute MI compared to the healthy controls, suggestive of endothelial dysfunction and this is consistent with prior findings in patients with other forms of CVD^17,18,35^. Slower coronary flow has previously been associated with atherosclerosis and increased risk of obstructive coronary artery disease^36^ and our findings would suggest a possible correlation between diminished epicardial blood flow and conjunctival Va. The most pronounced statistical differences for Va between the two study groups were observed for vessel size groups 2–4.

Overall blood volume flow (Q) did not differ significantly between the two study groups but was significantly lower in the MI group for vessel groups 3 and 4. These were the most numerically abundant vessel groups in the study. This may be explained by the distribution of venules and arterioles across the vessel size groupings. Differences in conjunctival Q was not reported in other studies of the conjunctival microcirculation in high CVD risk patients e.g. ischaemic stroke patients and diabetic patients^17,18^.

WSR was significantly reduced for the MI patients compared to the healthy controls, and this was consistent throughout all the vessel groups. Reduced WSR has previously been reported in patients at risk of CVD^15,18^. WSR has an important role in endothelial function^37,38^ and reduced WSR can lead to abnormal vascular wall remodelling^39^. Wall shear stress (WSS) is a direct product of WSR and plasma viscosity. Patients suffering type 1 acute MI have epicardial macrocirculatory disease^24^. Microcirculatory dysfunction, in the form of reduced WSS, has been reported in the coronary arteries of acute coronary syndrome patients with high-risk plaques^40^. Our findings would suggest that reduced WSR has a potential role in identifying those patients at high risk of MACE.

To our knowledge, at the time of writing, we are the only group to report findings and differences in conjunctival microcirculatory parameters for MI patients compared to healthy volunteers.

A potential limitation is that we studied the microcirculatory parameters without separating vessels into arterioles and venules. Differentiation between venules and arterioles has been reported previously based on the manually observed direction of flow within each vessel^10,11^. A major limitation of this method though is human error and the inability to reliably identify branching patterns. Automated differentiation of vessels into venules and arterioles would take require extensive technical development and was not possible within the confines of this pilot study. Haemoglobin video imaging (HVI) may also have the potential to help assess the conjunctival vessel hierarchy and vessel distribution^21^ but our methods would require further technical development to allow for integration into our model. Vessel differentiation may explain the statistical differences in blood volume flow that were only appreciated for group size 3–4 vessels. Another limitation is the inter-visit variability in conjunctival measurements. All MI patients and controls were recruited at one time-point. Previous studies have described the inter-visit variability of conjunctival measurements in both healthy controls^41^ and diabetic retinopathy patients^42^. The majority of the MI patients had received at least one dose of routinely prescribed medications (ACE inhibitor, beta-blocker) and all MI patients received dual antiplatelet therapy. These medications could contribute to the results observed between the two groups but the median time to recruitment post MI was 1 day and the vast majority would have only had a one-two doses of these drugs. As anticipated, there was a higher prevalence of diabetes mellitus, smoking history, systemic hypertension and dyslipidaemia in the MI group highlighting the severe CVD phenotype of MI patients. The subsequent sub-group analysis only demonstrated a significant difference in microcirculatory parameters for blood volume flow (Q) in patients with or without diabetes mellitus. This is a pilot investigation of MI patients conjunctival assessment and conventional sample size estimation was not feasible thus our findings are largely exploratory. Finally, vessels of smaller size were less abundant than the larger vessels but using our methods we still provided good results representation from the smaller vessel groups.

In conclusion this study using a slit-lamp biomicroscope and iPhone combination found lower axial velocity and wall shear rate in acute MI patients compared to healthy controls. In addition for MI patients, blood volume flow was lower in larger vessels compared to healthy controls. These results and the application of these methods have potential as an adjunct in cardiovascular risk assessment and screening which merit further development and study.

## Supplementary Information

Supplementary Information 1.

Supplementary Video S1.

## Data Availability

The datasets generated during and/or analysed during the current study are available from the corresponding author on reasonable request.
