# Supplementary material for: Assessment of the conjunctival microcirculation for patients presenting with acute myocardial infarction compared to healthy controls
Source: Sci Rep. 2021 Apr 7;11:7660. doi: 10.1038/s41598-021-87315-7 (PMC8027463; doi:10.1038/s41598-021-87315-7)
Supplement: Supplementary file 1 — Supplementary Information 1. [file 41598_2021_87315_MOESM1_ESM.docx]

**SUPPLEMENTARY MATERIAL**

**Manuscript title** Assessment of the conjunctival microcirculation for patients presenting with acute myocardial infarction compared to healthy controls

**Author list (in order)**

1. Dr Paul F. Brennan^1,2^

2. Dr Andrew J. McNeil^2^

3. Dr Min Jing^4^

4. Miss Agnes Awuah^2^

5. Miss Julie S. Moore^2^

6. Dr Jonathan Mailey^1^

7. Professor Dewar D. Finlay^4^

8. Professor Kevin Blighe^2^

9. Professor James A.D McLaughlin^4^

10. Dr M. Andrew Nesbit^2^

11. Professor EmanueleTrucco^3^

12. Professor Tara C.B. Moore^2^

13. Dr Mark S. Spence^1^

**Corresponding author** Dr Paul Brennan^1^ paul.brennan@belfasttrust.hscni.net

**Institutions** *^1^ Department of Cardiology, Royal Victoria Hospital, Belfast Health and Social Care Trust, Belfast, United Kingdom*

*^2^ Biomedical Sciences Research Institute, Ulster University, Coleraine, United Kingdom*

*^3^ VAMPIRE project, Computing (SSEN), University of Dundee, Dundee, United Kingdom*

*^4^ Nanotechnology and Integrated Bioengineering Centre (NIBEC), Ulster University, Jordanstown, United Kingdom*

**Supplementary Table 1.** Summary of conjunctival microcirculatory parameters for all vessel sizes for the STEMI and NSTEMI populations

| **Microcirculatory parameter** | **STEMI (n=21)** | **NSTEMI (n=38)** | **p value** |
| --- | --- | --- | --- |
| D, μm ±SD | 22.04 ±7.49 | 22.33 ±7.70 | 0.45 |
| Va, mm/s ±SD | 0.50 ±0.17 | 0.49 ±0.16 | 0.12 |
| Q, pl/s ±SD | 151 ±124 | 153 ±124 | 0.97 |
| WSR, s^-1^ ±SD | 149 ±89 | 144 ±88 | 0.08 |

D- Diameter. VA- Axial velocity. Q- Blood flow. WSR- Wall shear rate. SD- Standard deviation. STEMI- ST segment elevation myocardial infarction. NSTEMI- Non ST segment elevation myocardial infarction.

**Supplementary Figure 1a. Sub-group analysis of MI patients with respect to microvessel diameter (D)**


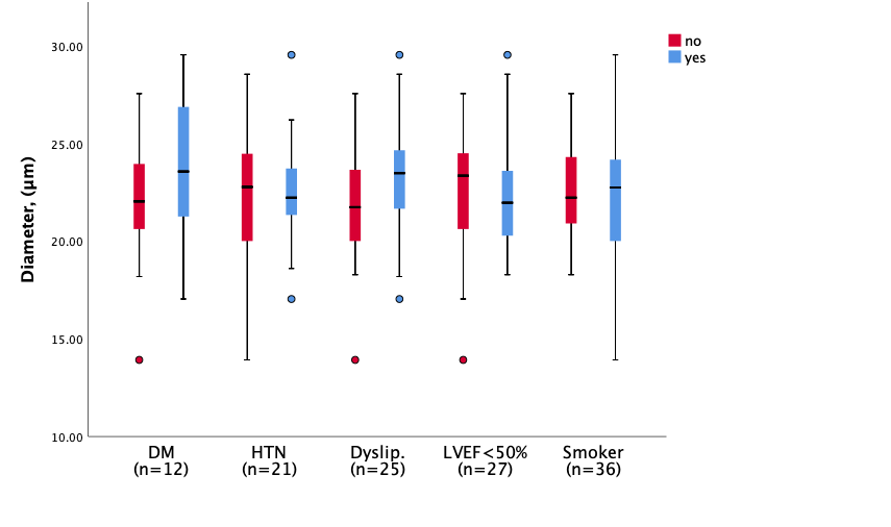


**Supplementary Video file 1. Processed and stabilised video of the left nasal conjunctival microvasculature in a healthy control.**

**Supplementary Figure 1b. Sub-group analysis of MI patients with respect to microvessel axial velocity (Va)**


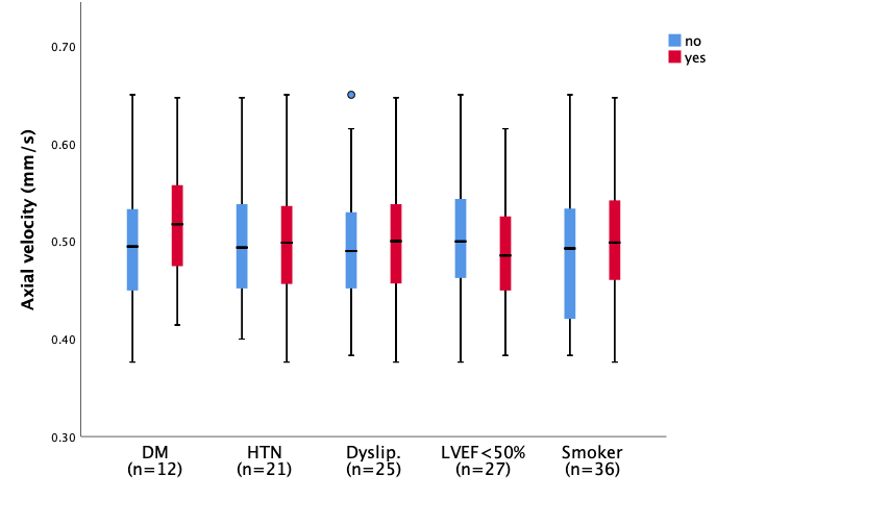


**Supplementary Figure 1c. Sub-group analysis of MI patients with respect to microvessel wall shear rate (WSR)**


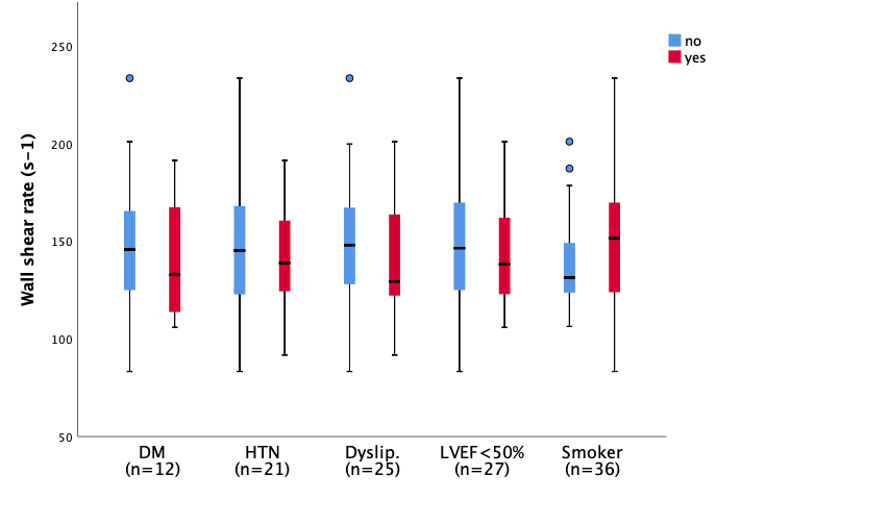


n denotes the number of “yes” for each sub-group.

DM- Diabetes Mellitus; HTN- Hypertension; Dyslip.- Dyslipidaemia; LVEF<50%- Left ventricular ejection fraction less than 50%; Smoker- Active smoker or Ex-smoker vs no history smoking.
